# The Na^+^/Ca^2+^ exchange inhibitor SEA0400 limits intracellular Ca^2+^ accumulation and improves recovery of ventricular function when added to cardioplegia

**DOI:** 10.1186/1749-8090-9-11

**Published:** 2014-01-08

**Authors:** Jeanne Egar, Ahmad Ali, Susan E Howlett, Camille Hancock Friesen, Stacy O’Blenes

**Affiliations:** 1 Department of Physiology and Biophysics, Dalhousie University, Halifax, Canada; 2 Department of Pharmacology, Halifax, Canada; 3 Department of Medicine, Div. of Geriatric Medicine, Halifax, Canada; 4 Department of Surgery, Halifax, Canada; 5 Department of Pathology, Halifax, Canada; 6 IWK Children’s Heart Centre, IWK Health Centre, 5850/5980 University Avenue, PO Box 9700, Halifax B3K 6R8, NS, Canada

**Keywords:** Myocardial protection/cardioplegia, Myocardium, Ischemia, Ischemia/reperfusion injury, Cardiac function

## Abstract

**Background:**

The Na^+^/Ca^2+^ exchange inhibitor SEA0400 prevents myocardial injury in models of global ischemia and reperfusion. We therefore evaluated its potential as a cardioplegia additive.

**Methods:**

Isolated rat cardiomyocytes were exposed to hypoxia (45 min) followed by reperfusion. During hypoxia, cells were protected using cardioplegia with (n = 25) or without (n = 24) SEA0400 (1 μM), or were not protected with cardioplegia (hypoxic control, n = 8). Intracellular Ca^2+^ levels were measured using Ca^2+^ sensitive dye (fura-2 AM). Isolated rat hearts were arrested using cardioplegia with (n = 7) or without (n = 6) SEA0400 (1 μM) then reperfused after 45 min of ischemia. Left ventricular (LV) function, troponin release, and mitochondrial morphology were evaluated.

**Results:**

Cardiomyocytes exposed to hypoxia without cardioplegia had poor survival (13%). Survival was significantly improved when cells were protected with cardioplegia containing SEA0400 (68%, p = 0.009); cardioplegia without SEA0400 was associated with intermediate survival (42%). Cardiomyocytes exposed to hypoxia alone had a rapid increase in intracellular Ca^2+^ (305 ± 123 nM after 20 minutes of ischemia). Increases in intracellular Ca^2+^ were reduced in cells arrested with cardioplegia without SEA0400; however cardioplegia containing SEA0400 was associated with the lowest intracellular Ca^2+^ levels (110 ± 17 vs. 156 ± 42 nM after 45 minutes of ischemia, p = 0.004). Hearts arrested with cardioplegia containing SEA0400 had better recovery of LV work compared to cardioplegia without SEA0400 (23140 ± 2264 vs. 7750 ± 929 mmHg.μl, p = 0.0001). Troponin release during reperfusion was lower (0.6 ± 0.2 vs. 2.4 ± 0.5 ng/mL, p = 0.0026), and there were more intact (41 ± 3 vs. 22 ± 5%, p < 0.005), and fewer disrupted mitochondria (24 ± 2 vs. 33 ± 3%, p < 0.05) in the SEA0400 group.

**Conclusions:**

SEA0400 added to cardioplegia limits accumulation of intracellular Ca^2+^ during ischemic arrest in isolated cardiomyocytes and prevents myocardial injury and improves recovery of LV function in isolated hearts.

## Background

Myocardial ischemia-reperfusion injury is mediated by influx of Ca^2+^ into cardiomyocytes leading to cardiomyocyte injury or death through mechanisms including hypercontracture [1-3], and mitochondrial damage and dysfunction [4,5]. The Na^+^/Ca^2+^ exchanger (NCX) plays an essential role in the pathophysiology of Ca^2+^ accumulation during ischemia [6]. Its normal function is to remove Ca^2+^ from the cytoplasm in exchange for Na^+^. However, intracellular acidosis resulting from anaerobic metabolism during ischemia causes Na^+^ influx through the Na^+^/H^+^ exchanger and Na^+^/HCO_3_^–^ symporter [3,6,7]. Increased intracellular Na^+^ drives reverse mode action of NCX, which brings Ca^2+^ into the cell [6]. Cardioplegia solutions reduce myocardial metabolism by inducing electromechanical arrest typically with hyperkalemia, and often combined with hypothermia to slow the development of intracellular acidosis responsible for Na^+^, and subsequently Ca^2+^ influx via reverse mode NCX activity [6]. Cardioplegia has improved the results of cardiac surgery but the duration of ischemia is still a predictor of impaired myocardial function and death after cardiac surgery, suggesting that current myocardial protection strategies can be improved [8].

SEA0400 is a potent inhibitor of NCX with preferential blockade of reverse mode function (Ca^2+^ influx) and limited impact on L-type Ca^2+^ channels at concentrations ≤1 μM [9,10]. In experimental models, SEA0400 prevents cytoplasmic and mitochondrial Ca^2+^ overload during ischemia [11-13] and results in improved recovery of ventricular function after reperfusion [13,14] associated with mitochondrial preservation [11,13]. However, because NCX activity is determined by membrane potential and trans-membrane ion gradients [15-17], it is not clear if these observations are generalizable in the context of hyperkalemic cardioplegic arrest. We therefore tested the hypothesis that blockade of NCX with the addition of SEA0400 to a cardioplegia solution would limit accumulation of intracellular Ca^2+^ during ischemia and result in superior recovery of left ventricular (LV) function after reperfusion.

## Methods

### Animals

Experiments followed guidelines of the Canadian Council on Animal Care (CCAC; Ottawa, ON: Vol. 1, 2^nd^ edition, 1993; Vol. 2, 1984). Male Fisher rats (≈3 months, ≈300 g, Charles River Canada, St. Constant, Canada) were heparinized (3000 U/kg intraperitoneal; Pharmaceutical Partners of Canada, Richmond, Canada) and anaesthetized (sodium pentobarbital 160 mg/kg intra-peritoneal; CDMV; Saint-Hyacinthe, Canada) prior to harvest of hearts or cardiomyocytes and were approved by the Dalhousie University Animal Care Committee.

### Cardioplegic arrest - isolated cardiomyocytes

Ventricular myocytes were obtained by enzymatic dissociation according to previously described methods [18,19]. Briefly, hearts were perfused *in situ* for 5 min with buffer containing (mM): 135.5 NaCl, 4 KCl, 10 HEPES, 1.2 MgSO_4_, 1.2 KH_2_PO_4_ 12 glucose and 200 μM CaCl_2_ (pH 7.4, 37°C, 100% O_2_). Hearts were then perfused with this solution without CaCl_2_ (5 min), followed by (20 min) buffer containing 50 μM CaCl_2_, protease dispase II (0.1 mg/mL, Roche Diagnostics, Laval, Canada), collagenase type 2 (0.56 mg/mL, Worthington, Lakewood, NJ) and trypsin (0.02 mg/mL, Sigma-Aldrich, Oakville, Canada). The ventricles were minced in buffer containing (mM): 45 KCl, 3 MgSO_4_.7H2O, 30 KH_2_PO_4_, 50 L-glutamic acid, 20 taurine, 0.5 EGTA, 10 HEPES and 10 glucose (pH 7.4 with KOH). Quiescent, rod shaped, cardiomyocytes with no visible membrane damage were used. A maximum of two cardiomyocytes per heart were used in any experimental group.

Individual cardiomyocytes were placed in a chamber on an inverted microscope and superfused with buffer containing (mM): 126 NaCl, 20 NaHCO_3_, 0.9 NaH_2_PO_4_, 4 KCl, 0.5 MgSO_4_, 5.5 glucose and 1.8 CaCl_2_ (pH 7.4, 37°C, 95% O_2_, 5% CO_2_). After equilibration (20 min), cells were superfused with buffer formulated to simulate the cardioplegia we use in the clinic containing (mM): 118 Na^+^, 18 K^+^, 5 Mg^+^, 1.0 Ca^2+^, and SEA0400 (1 μM, Taisho Pharmaceutical Co, Ltd., Tokyo, Japan, n = 25) or its vehicle, DMSO (0.1%, n = 24). The O_2_ scavenger sodium dithionite (5 mM) was added to the cardioplegia and it was bubbled with 90% N_2_ and 10% CO_2_ and this gas was directed over the chamber during the cardioplegia period to reduce the extracellular pO_2_ to ≈ 12 mmHG and pH was reduced to 6.8 to simulate conditions at the tissue level during cardioplegic arrest [20,21]. Cardioplegia used in the isolated cell studies did not contain blood. Following cardioplegic arrest (45 min), cardiomyocytes were reperfused with oxygenated buffer. Ischemic control cells were exposed to hypoxia but not the cardioplegia solution. Myocytes were field stimulated (1 Hz) throughout the protocol. Cell death was identified visually when cells lost the typical cardiomyocyte morphology and rounded up into a ball, and was confirmed by trypan blue staining.

### Measurement of intracellular Ca^2+^ and cardiomyocyte contraction amplitude

At the start of the experimental protocol, cells were loaded with the Ca^2+^ sensitive dye fura-2 AM (5 μM, 20 min, RT, Invitrogen, Burlington, Canada) and intracellular Ca^2+^ was measured by whole cell photometry (DeltaRam, Photon Technology International, Birmingham, NJ) according to previously described techniques [18,19]. The emission ratio at 510 nm, during alternate excitation at 340 and 380 nm was used to determine intracellular Ca^2+^ concentrations. Background fluorescence was determined at each excitation wavelength and subtracted from the recordings. Emission ratios were converted to intracellular Ca^2+^ concentrations using an *in-vitro* calibration curve. Unloaded cell shortening was measured with a video edge detector (Crescent Electronics, Sandy, UT). Ten second trains of contractions were averaged and measured with Clampfit 8.2 (Molecular Devices, Sunnyvale, CA). Contraction amplitude is the difference between systolic and diastolic cell length. All values were normalized to the time point immediately prior to ischemia.

### Cardioplegic arrest – isolated hearts

Rats were assigned to their experimental group before the experiment started. Hearts were attached by the aorta to a Langendorff apparatus (AD Instruments Inc., Colorado Springs, CO). Shed blood was collected for mixing with the cardioplegia solution. Electrocardiogram and aortic pressure were recorded continually. Coronary flow was measured using a transit time ultrasound probe (Transonic Systems Inc., Ithica, NY) on the inflow cannula, and coronary vascular resistance was calculated by dividing aortic pressure by flow. A custom non-compliant balloon tipped catheter was placed through the mitral valve into the LV cavity. Hearts were perfused with oxygenated Kreb’s solution (37ºC, 10 mL/min, [Ca^2+^] = 2.5 mM) in a heated chamber (20 min) then baseline LV function data were obtained (see below). Perfusion was interrupted and cardioplegia delivered through the aorta (20 mL/kg body weight). Cardioplegia was prepared by mixing base solution used in clinical practice (76.1 mM KCl, 20.2 mM MgSO_4_, 86.5 mM NaHCO_3_ in 1 L 5% Dextrose/0.225% NaCl) with the shed blood in a ratio of 4 parts blood to 1 part base solution resulting in a final cardioplegia solution with an approximate ionic composition of (mM): 136 Na^+^, 19 K^+^, 5 Mg^2+^, and 1 Ca^2+^. SEA0400 (1 μM, n = 7) or its vehicle (DMSO 0.1%, n = 6) was added to the cardioplegia, which was cooled on an ice bath (≈4°C) and bubbled with oxygen prior to delivery. After cardioplegia delivery, the heart was exposed to ambient room temperature (22 ± 0.2°C) for the duration of the ischemic period (45 min). After reperfusion with 37°C oxygenated Kreb’s solution (20 min), LV function data were again collected.

### LV function

Load independent indices were derived from LV pressure recordings during incremental inflation of the intra-ventricular balloon in 25 μL steps to a maximum of 200 μL according to the method described by Li et al. [22]. End-systolic and end-diastolic pressures were plotted against LV volume and the pressure-volume relationships estimated by linear regression. LV work, a load-independent index of ventricular function, was estimated by the area between the systolic and diastolic pressure-volume relationships [22]. LV developed pressure, dP/dt+, and dP/dt- were also recorded.

### Reperfusion arrhythmias

The electrocardiogram was recorded continuously throughout the experiment using epicardial electrodes. The start time, end time, and incidence of ventricular arrhythmias (tachycardia and fibrillation) during reperfusion were quantified.

### Myocardial and mitochondrial injury

Myocardial damage was assessed by measuring troponin I release from timed collections of coronary sinus effluent during reperfusion (ELISA assay, Life Diagnostics, West Chester, PA). Mitochondria were isolated from LV muscle and a blinded observer categorized and counted intact, swollen, and disrupted mitochondria from random electron photomicrographs from each heart [23,24].

### Statistical analysis

Data are presented as mean ± SEM. Tests for statistical significance included Kaplan-Meier log-rank survival analysis, unpaired t-test, and one-way and two-way repeated measures ANOVA with post-hoc comparisons by Newman-Keuls and Holmes-Sidak method respectively.

## Results

### Cardioplegia containing SEA0400 improved cardiomyocyte survival during ischemia

**Figure 1:**
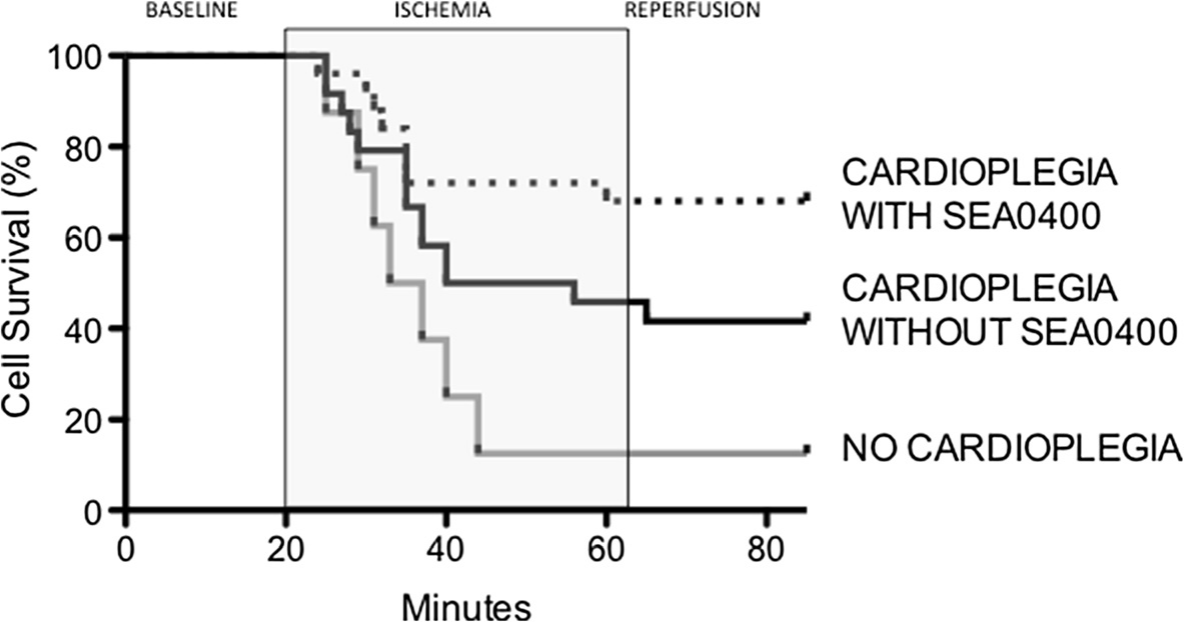
**Effect of cardioplegia containing SEA0400 on cell survival in isolated cardiomyocytes during ischemia and reperfusion.** Lines represent the proportion of cells surviving at each time point throughout the experimental protocol. Cell Survival was higher in cells protected with cardioplegia containing SEA0400 when compared to cells exposed to ischemia alone (no cardioplegia, p = 0.009).

We examined the impact of simulated cardioplegia containing the NCX blocker SEA0400 on cell survival during ischemia and reperfusion in isolated cardiomyoyctes (Figure 1). Cell survival was poor (1 of 8, 13%) in cells exposed to ischemia without cardioplegia. Arrest with simulated cardioplegia containing SEA0400 was associated with significantly higher survival during ischemia (17 of 25, 68%, p = 0.009 vs. ischemia alone). Cells arrested with cardioplegia not containing SEA0400 had an intermediate survival rate (10 of 24, 42%, p = NS vs. ischemia alone and vs. cardioplegia with SEA0400).

### Addition of SEA0400 to cardioplegia reduced intracellular Ca^2+^ accumulation during ischemia

**Figure 2:**
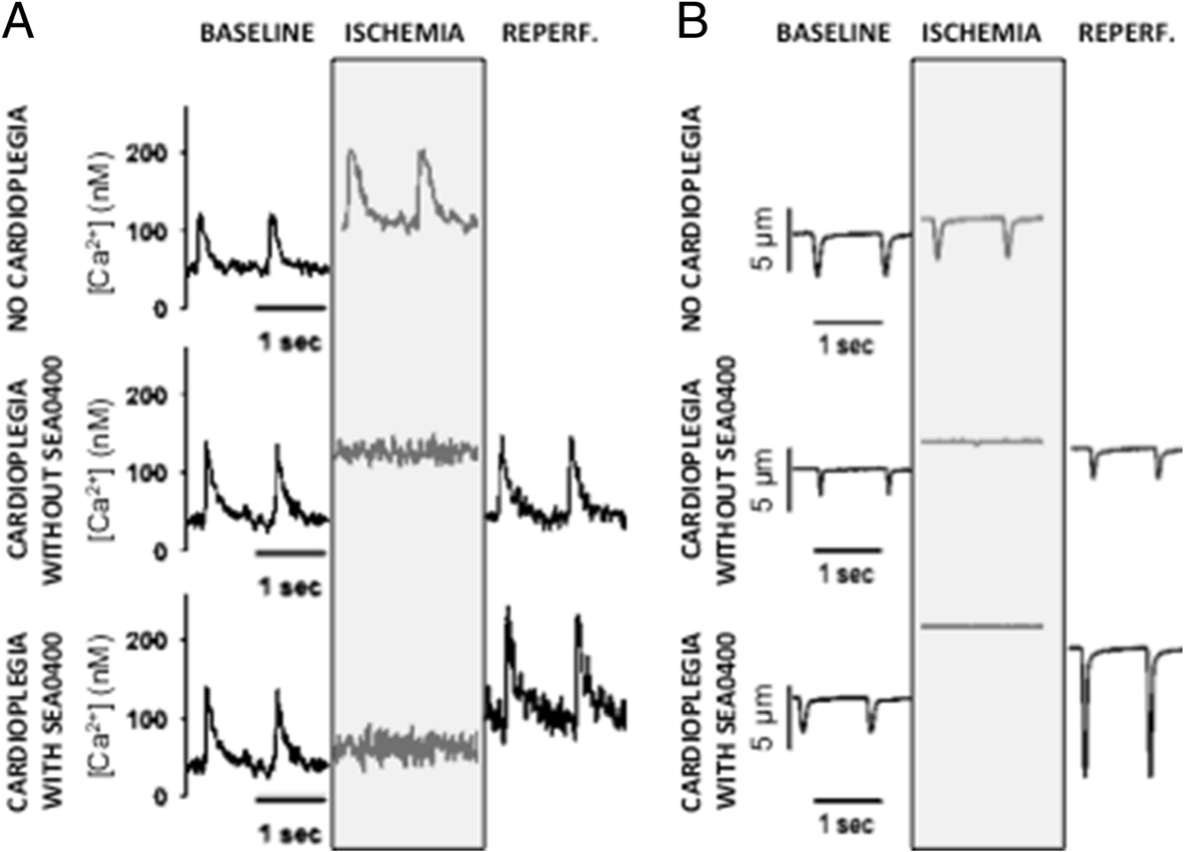
**Effect of SEA0400 in cardioplegia on intracellular Ca**^**2+ **^**and cell shortening in isolated cardiomyocytes. A)** Representative recordings of intracellular Ca^2+^ at baseline, during ischemia, and after reperfusion in cardiomyocytes arrested using cardioplegia with or without SEA0400, or exposed to ischemia alone (no cardioplegia). **B)** Representative recordings of cell shortening acquired simultaneously with the intracellular Ca^2+^ measurements.

**Figure 3:**
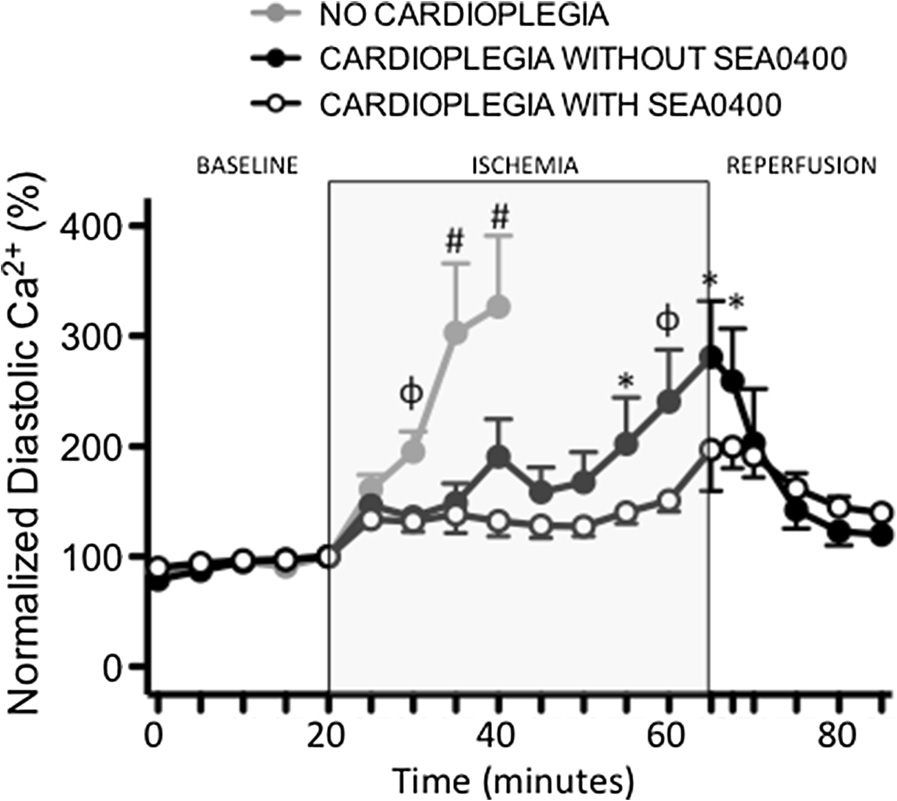
**Effect of SEA0400 in cardioplegia on diastolic Ca**^**2+ **^**in isolated cardiomyocytes.** Graph of intracellular diastolic Ca^2+^ measured using fluorescent Ca^2+^ sensitive dye. Points represent mean ± SEM, n = 25 (cardioplegia with SEA0400), 24 (cardioplegia without SEA0400), and 8 (no cardioplegia). All values are normalized to the time point immediately prior to ischemia (20 min). * = p < 0.05, ϕ = <0.01, # = p < 0.001 vs. cardioplegia with SEA0400.

**Figure 4:**
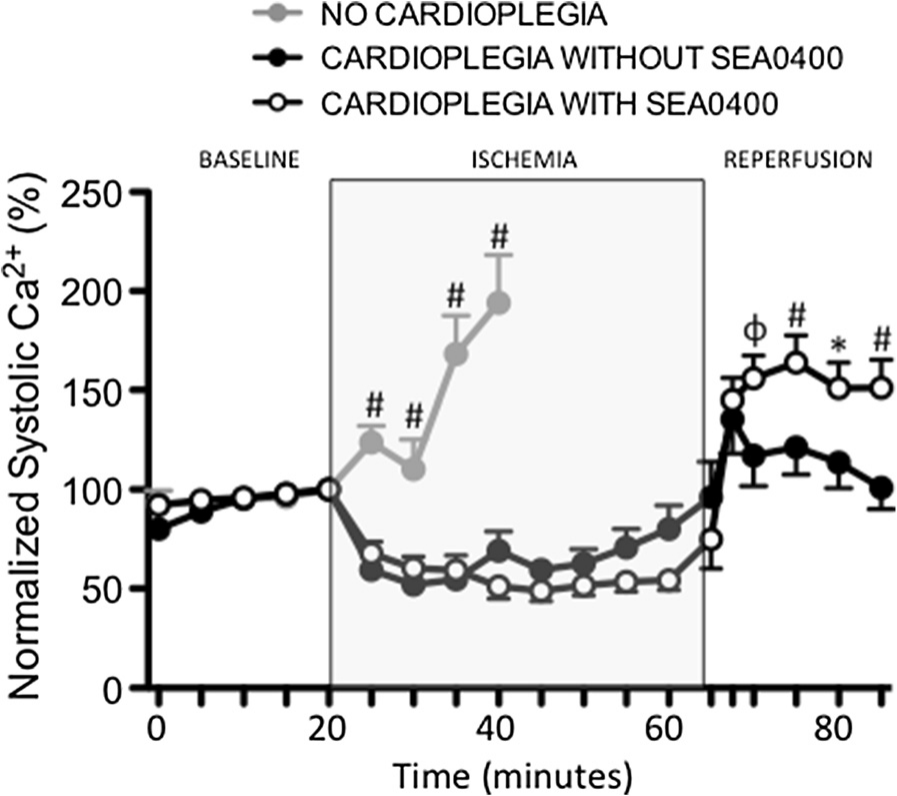
**Effect of SEA0400 in cardioplegia on systolic Ca**^**2+ **^**in isolated cardiomyocytes.** Graph of intracellular systolic Ca^2+^ measured using fluorescent Ca^2+^ sensitive dye. Points represent mean ± SEM, n = 25 (cardioplegia with SEA0400), 24 (cardioplegia without SEA0400), and 8 (no cardioplegia). All values are normalized to the time point immediately prior to ischemia (20 min). * = p < 0.05, ϕ = <0.01, # = p < 0.001 vs. cardioplegia with SEA0400.

**Figure 5:**
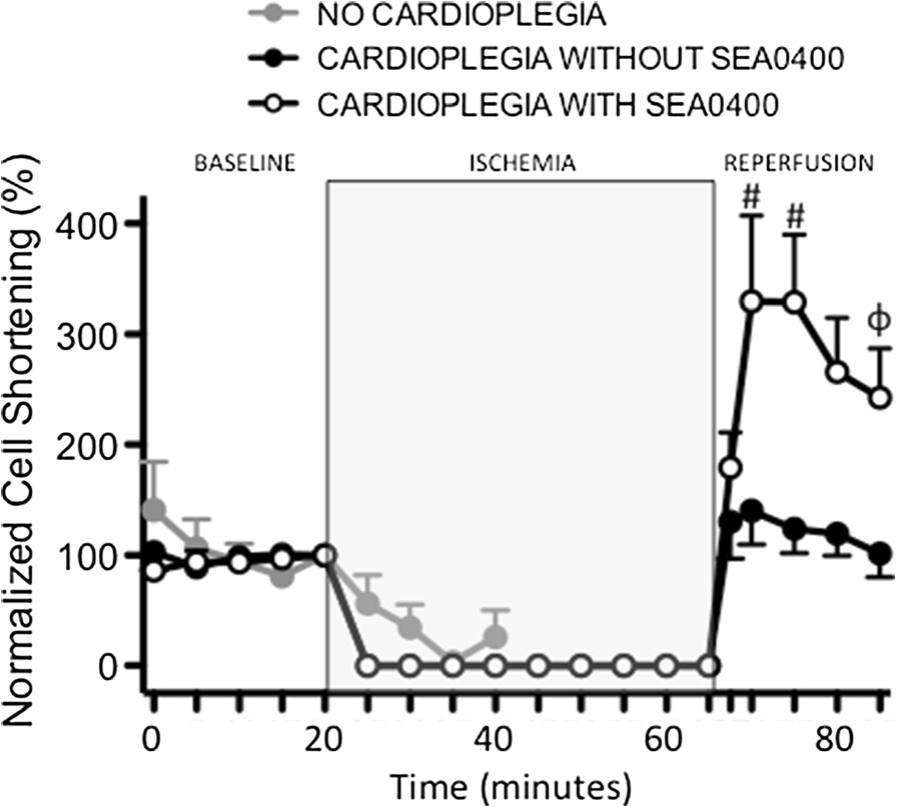
**Effect of SEA0400 in cardioplegia on cell shortening in isolated cardiomyoyctes.** Graph of cell shortening (contraction amplitude) measured using video edge detection. Points represent mean ± SEM, n = 25 (cardioplegia with SEA0400), 24 (cardioplegia without SEA0400), and 8 (no cardioplegia). All values are normalized to the time point immediately prior to ischemia (20 min)*. ϕ = <0.01, # = p < 0.001 vs. cardioplegia with SEA0400.*

Intracellular Ca^2+^ levels were measured in isolated cardiomyocytes during ischemia and reperfusion. Ischemia without cardioplegic protection was associated with a rapid increase in both diastolic and systolic intracellular Ca^2+^ levels (Figures 2, 3 and 4). Diastolic Ca^2+^ levels rose from 77 ± 9 nM at baseline to 305 ± 123 nM (≈300%) by 20 minutes into the ischemic period (p < 0.001). Intracellular Ca^2+^ accumulation was limited in cells arrested with cardioplegia (Figures 2A and 3). Diastolic Ca^2+^ rose ≈ 180% from 56 ± 5 nM at baseline to 156 ± 42 nM at the end of the ischemic period in cells protected with cardioplegia not containing SEA0400 (p < 0.001). Diastolic Ca^2+^ levels rose least in cells protected with cardioplegia containing SEA0400 from 62 ± 5 nM at baseline to 110 ± 17 nM (77%) at the end of the ischemic period (p = <0.001 vs. baseline, p < 0.004 vs. cardioplegia without SEA0400). While diastolic Ca^2+^ levels returned towards baseline values soon after reperfusion in both groups protected with cardioplegia (Figures 2A and 3), systolic Ca^2+^ levels were higher during the reperfusion period in cells protected with cardioplegia containing SEA0400 (Figures 2A and 4) resulting in larger contractions (Figures 2B and 5)

### SEA0400 in cardioplegia improved recovery of LV function after ischemic cardioplegic arrest

**Figure 6:**
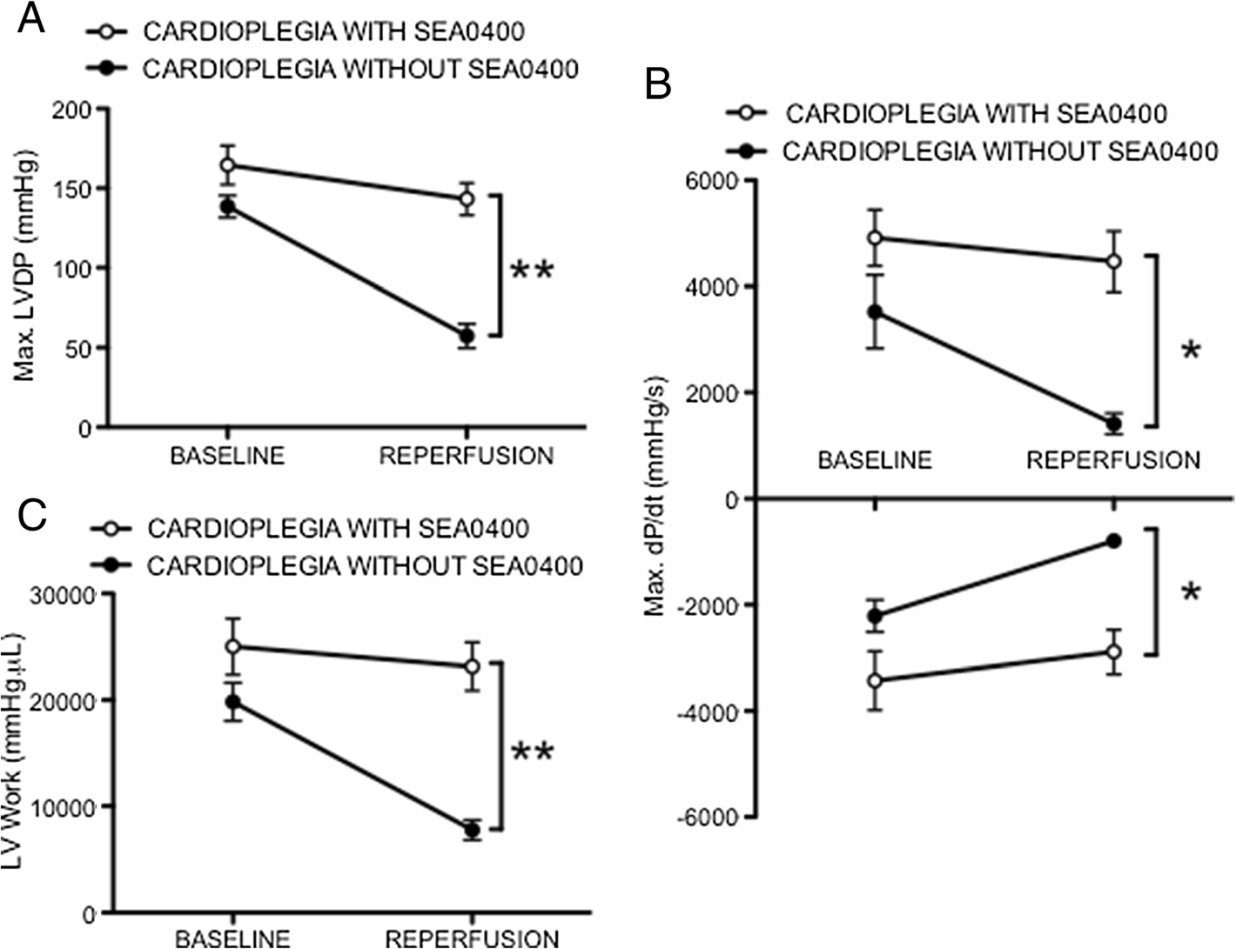
**Effect of SEA0400 in cardioplegia on recovery of LV function in isolated hearts. A)** Maximum LV developed pressure (LVDP) determined from the intra-ventricular balloon recordings before arrest (baseline) and after reperfusion. **B)** Maximum dP/dt + and dP/dt-. **C)** LV work estimated by the area between the end-systolic and end-diastolic pressure-volume relationships. Data points represent mean ± SEM, * = p < 0.001, ** = p < 0.0001 for cardioplegia wihout SEA0400 (n = 6) vs. cardioplegia with SEA0400 (n = 7). Baseline values for the two groups were not significantly different.

To evaluate the impact of SEA0400 as a cardioplegia additive on recovery of LV function, hearts were subjected to 45 minutes of ischemia after arrest induced by cold blood cardioplegia with or without SEA0400. There was no spontaneous electromechanical activity observed in either group during the ischemic period. There were no significant differences in baseline functional parameters between groups. Maximum LV developed pressure after reperfusion recovered to 57 ± 8 mmHg (43% of baseline) in hearts arrested with cardioplegia not containing SEA0400 (Figure 6A). However, when SEA0400 was added to the cardioplegia, recovery of maximum LV developed pressure was substantially larger (143 ± 10 mmHg, 88% of baseline, p = 0.0001). Similarly, recovery of maximum dP/dt + and dP/dt- were both significantly greater in hearts protected with cardioplegia containing SEA0400 (4465 ± 573 vs. 1409 ± 198 mmHg/s, p = 0.0006 for dP/dt + and 2890 ± 417 vs. 793 ± 109 mmHg/s, p = 0.0009 for dP/dt-, Figure 6B). LV work in the hearts arrested with cardioplegia not containing SEA0400 recovered to 7750 ± 929 mmHg.μL (39% of baseline) but was markedly higher in hearts protected with cardioplegia containing SEA0400 (23140 ± 2264 mmHg.μL, 93% of baseline, p = 0.0001, Figure 6C).

### Reperfusion arrhythmias were not increased in hearts arrested with cardioplegia containing SEA0400

We quantified reperfusion arrhythmias (ventricular tachycardia and fibrillation) after cardioplegic arrest. All hearts had one or more episodes of ventricular arrhythmia. All episodes terminated spontaneously. The onset of reperfusion arrhythmias was delayed in the SEA0400 group (342 ± 93 vs. 100 ± 7 s, p = 0.036) but the resolution of arrhythmias occurred at a similar time in each group (without SEA0400 1029 ± 73 s, with SEA0400 940 ± 102 s). The number of distinct episodes of arrhythmia was 16 ± 3 episodes in the group not treated with SEA0400 and 7 ± 3 episodes in the SEA0400 group, a difference that may have been due to chance (p = 0.054).

### SEA0400 in cardioplegia prevented myocardial damage after ischemic cardioplegic arrest and reperfusion

**Figure 7:**
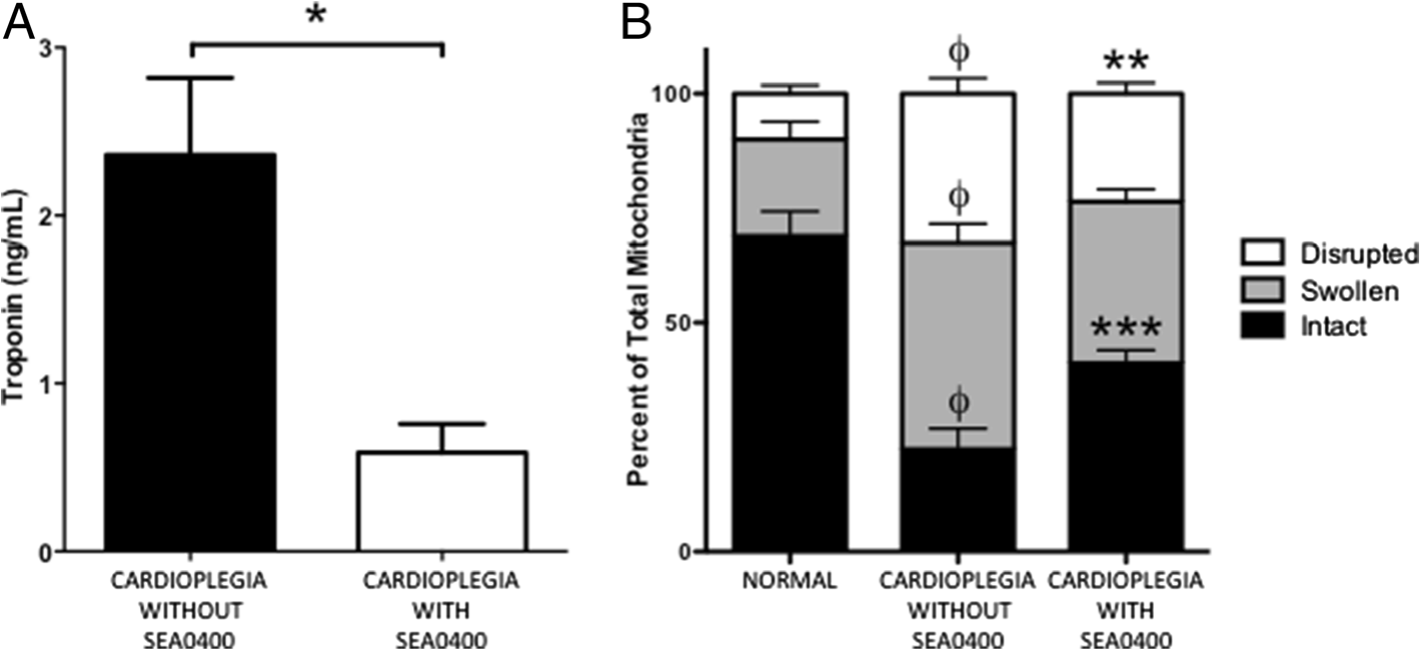
**Effect of SEA0400 in cardioplegia on troponin release and mitochondrial morphology in isolated hearts. A)** Troponin release was measured by ELISA assay from coronary sinus effluent during reperfusion. **B)** Relative number of intact, swollen, and disrupted mitochondria was quantified from electron micrographs of isolates from LV myocardium after cardioplegic arrest and reperfusion. Mitochondria isolated from hearts not subjected to ischemic cardioplegic arrest (Normal) were analyzed for comparison. Bars represent mean ± SEM, * = p = 0.0026 for cardioplegia without SEA0400 vs. cardioplegia with SEA0400, Ф = p < 0.001 for Normal vs. cardioplegia without SEA0400, ** = p < 0.05 for disrupted mitochondria (cardioplegia without vs. with SEA0400), *** = p < 0.01 for intact mitochondria (cardioplegia without vs. with SEA0400), n = 6 (Normal & cardioplegia without SEA0400), and 7 (cardioplegia with SEA0400).

Troponin release during reperfusion was reduced in hearts protected with cardioplegia containing SEA0400 when compared to those without SEA0400 (0.6 ± 0.2 vs. 2.4 ± 0.5 ng/mL, p = 0.0026, Figure 7A). Coronary vascular resistance during reperfusion, an indicator of vascular dysfunction and myocardial edema, increased to 183 ± 23% of baseline (9.0 mmHg.min.ml^-1^) in hearts arrested with cardioplegia without SEA0400 but was only 109 ± 9% of baseline (7.0 mmHg.min.ml^-1^) in hearts protected with cardioplegia containing SEA0400 (p = 0.0088). The proportion of intact mitochondria was lower and the proportion of disrupted mitochondria was higher in isolates from LV myocardium after cardioplegic arrest and reperfusion when compared to normal myocardium not subjected to ischemia (Figure 7B). However, in those hearts treated with cardioplegia containing SEA0400, there were more intact mitochondria (41 ± 3 vs. 22 ± 5%, p < 0.01) and fewer disrupted mitochondria (24 ± 2 vs. 33 ± 3%, p < 0.05) when compared to those treated with cardioplegia not containing SEA0400 (Figure 7B).

## Discussion

The objective of our study was to examine the impact of SEA0400 on intracellular Ca^2+^ levels during cardioplegic arrest and determine if SEA0400 enhances myocardial protection when added to cardioplegia. We found that SEA0400 limits the accumulation of intracellular Ca^2+^ during ischemia when added to cardioplegia. The addition of SEA0400 to cardioplegia resulted in superior protection against myocardial and mitochondrial injury and was associated with improved recovery of LV function.

### Global ischemia vs. Cardioplegic arrest

Reverse mode action of NCX during ischemia leading to Ca^2+^ influx, hypercontracture, and mitochondrial damage is felt to be the primary mechanism responsible for myocardial injury in ischemia-reperfusion [1-7]. As such, SEA0400 is an attractive cardioplegia additive based on its preferential block of reverse mode NCX activity [9,10]. While SEA0400 has been extensively investigated in models of global ischemia [10-14], it has not previously been investigated in the setting of cardioplegic arrest. Global ischemia is associated with dramatic increases in intracellular Ca^2+^ driven by the development in intracellular acidosis [1,3]. While the underlying mechanism of cell injury in the context of cardioplegic arrest may be similar, there are several factors that may limit the generalizability of observations from the global ischemia model to cardioplegic arrest. The aim of cardioplegic arrest is to minimize metabolic activity in the cardiomyocyte thereby delaying the development of intracellular acidosis, which is the driver for Ca^2+^ influx. Indeed, we observed that intracellular Ca^2+^ levels increased much faster in cells not protected with cardioplegia. However, the relative importance of NCX activity in Ca^2+^ influx may also differ between the conditions of global ischemia versus cardioplegic arrest. The direction and magnitude of Ca^2+^ exchange is related to membrane potential, which is affected by cardioplegia, and the transmembrane Ca^2+^ gradient [15-17] which may also be affected by cardioplegia solutions which often have reduced Ca^2+^ concentrations. Therefore, we felt it was important to evaluate a strategy of NCX blockade specifically in the context of cardioplegic arrest.

### SEA0400 as a cardioplegia additive

In isolated cardiomyocytes, we found that simulated cardioplegia limited the development of intracellular Ca^2+^ overload. However, diastolic Ca^2+^ levels still rose above baseline values by the end of the 45 minute ischemic period. In cells arrested with cardioplegia containing SEA0400, Ca^2+^ overload was further reduced supporting the concept that reverse mode NCX exchange does play a role in Ca^2+^ homeostasis in the cardioplegia-arrested cell. This was associated with evidence of improved myocardial protection in the whole heart including lower troponin release during reperfusion, preserved mitochondrial morphology, and better functional recovery. We hypothesize that in the whole heart, the addition of SEA0400 to cardioplegia prevents death of a proportion of cardiomyocytyes resulting in the lower troponin release observed. Furthermore, limitation of Ca^2+^ overload protects the mitochondria in surviving cells as evidenced by preserved mitochondrial morphology. These two features may both contribute to improved functional recovery in hearts arrested with cardioplegia containing SEA0400. In isolated cardiomyocytes we also found that while diastolic Ca^2+^ levels returned quickly to baseline values during the reperfusion period, systolic Ca^2+^ levels were somewhat higher in the cells protected with cardioplegia containing SEA0400. The cause for this is not clear but one possibility includes a persistent blockade of NCX during reperfusion. While SEA0400 preferentially blocks the reverse mode of NCX, it also blocks the forward mode to some degree [8,25] which, during reperfusion, may impede NCX’s normal physiologic function of removing Ca^2+^ from the cell after each contraction cycle. This could favor reuptake of Ca^2+^ by the sarcoplasmic reticulum resulting in larger amplitude Ca^2+^ induced Ca^2+^ release and higher systolic Ca^2+^ levels seen. While very high intracellular Ca^2+^ levels during reperfusion can lead to damaging hypercontracture, we did not see this in these cells. The higher systolic Ca^2+^ levels seen during reperfusion in cells protected with cardiolpegia containing SEA0400 resulted in larger contractions but no evidence of reduced diastolic cell length (data not shown).

We examined only one dose level of SEA0400 (1 uM), which was chosen based on previous studies that demonstrated relatively specific targeting of the reverse mode NCX activity (Ca^2+^ influx) at or below this concentration [9,10,25]. While a higher concentration of the drug might have resulted in further reductions of in Ca^2+^ influx during ischemia, potential blockade of forward mode NCX or unwanted effects on other ion channels (L-type Ca^2+^) during reperfusion may be problematic.

We focused on delivery of SEA0400 in the cardioplegia because Ca^2+^ influx is thought to occur as a result of reverse mode NCX activity driven by increased intracellular Na^+^ that develops during the ischemic period. Furthermore, this strategy would reduce the potential for unwanted remote side effects by minimizing systemic delivery.

### Arrhythmias with SEA0400

Hearts are susceptible to arrhythmia upon reperfusion after an ischemic period. A single report has described increased reperfusion arrhythmias after global warm ischemia in isolated hearts treated with SEA0400 [26]. We found that the onset of reperfusion arrhythmias was delayed when SEA0400 was added to cardioplegia. This may be related to a protective effect of SEA0400 that is ‘washed out’ of the myocardium during reperfusion. We saw no evidence for increased reperfusion arrhythmias, consistent with most reports [14,27,28].

### Limitations

The results of this study must be interpreted in the context of several important limitations. For technical reasons, isolated cardiomyocyte studies were performed using simulated cardioplegia that contained no blood and was delivered at normothermic temperatures which is a deviation from the usual clinical situation. For the isolated heart studies, we attempted to model the clinical scenario as closely as possible and were able to assess cardiac function in the short term using the Langendorff system; however, it was not feasible to study hearts at more clinically relevant time points (12 or 24 hours post reperfusion). While ischemia-reperfusion injury is primarily initiated by Ca^2+^ overload, other processes affect the development of myocardial injury and evolution of functional recovery. For example, the inflammatory system plays a role in the myocardial damage that occurs early after reperfusion [29]. Future studies in an intact animal model will be required to address these limitations.

## Conclusions

In isolated cardiomyocytes, SEA0400 limits Ca^2+^ overload during cardioplegic arrest. This is associated with reduced myocardial and mitochondrial damage and improved recovery of LV function in the whole heart. SEA0400 does not appear to increase reperfusion arrhythmias when used as a cardioplegia additive. Blockade of NCX activity with SEA0400 in cardioplegia is a promising strategy to improve myocardial protection during cardiac surgery and warrants further study to confirm the mechanism of action and to evaluate its benefit in human cells and a whole animal preclinical model.

## Abbreviations

LV: Left ventricle; NCX: Na^+^/Ca^2+^ exchanger; ELISA: Enzyme-linked immunosorbent assay; LVDP: Left ventricular developed pressure.

## Competing interests

SEA0400 used in this study was donated by Taisho Pharnaceutical Co, Ltd., Tokyo, Japan. The authors had full control of the design of the study, methods used, outcome parameters and results, analysis of data, and production of the written report. The authors have no financial interests to disclose.

## Authors’ contributions

JE carried out isolated cardiomyocyte studies. These were performed in the lab of SH. AA carried out the isolated heart studies. All authors were involved in the design of the studies. SO coordinated the study and helped to draft the manuscript. All authors read and approved the final manuscript.
